# The correlation between follow-up MRI findings and laboratory results in pyogenic spondylodiscitis

**DOI:** 10.1186/s12891-020-03446-4

**Published:** 2020-07-02

**Authors:** Kyung-Sik Ahn, Chang Ho Kang, Suk-Joo Hong, Baek Hyun Kim, Euddeum Shim

**Affiliations:** 1 Department of Radiology, Korea University Anam Hospital, Korea University College of Medicine, 73, Inchon-ro, Seongbuk-gu, Seoul, 02841 Korea; 2 Department of Radiology, Korea University Guro Hospital, Seoul, Korea; 3 Department of Radiology, Korea University Ansan Hospital, Gyeonggi-do, Korea

**Keywords:** Spine, Infection, Magnetic resonance imaging, C-reactive protein, Erythrocyte sedimentation rate

## Abstract

**Background:**

Although MRI is the gold-standard imaging method in the diagnosis of spondylodiscitis, role of follow-up imaging is debated and there can be discrepancies with regard to the significance of bony or soft tissue responses to treatment. Purpose of our study is to test whether the MRI changes on follow-up imaging correlate with laboratory findings of treatment response.

**Methods:**

A total of 48 patients with pyogenic spondylodiscitis who underwent baseline and follow-up MRI were retrospectively reviewed. The extent of bone marrow edema, paravertebral soft tissue inflammation, and disc height were compared on baseline and follow-up MRIs with the C-reactive protein (CRP) and erythrocyte sedimentation rate (ESR) levels obtained from the medical records at baseline and on follow-up. Relationships between the MRI and laboratory changes were analyzed using the Spearmann correlation test.

**Results:**

The mean MRI follow-up period was 42.25 days. Based on the CRP (resolved: *n* = 19, resolving: n = 19, and aggravated: *n* = 10), there was significant correlation between the laboratory results and the changes in the bone and soft tissues (*p* < 0.01, both). The correlation was best with soft tissue changes (rho: 0.48) followed by bony changes (rho: 0.41). Based on the ESR (resolved: *n* = 8, resolving: *n* = 22, and worsened: *n* = 18), the correlation was stronger with bone changes (rho: 0.45, *p* < 0.01) than it was with soft tissue changes (rho: 0.39, *p* = 0.01).

**Conclusion:**

Follow-up MRI findings of pyogenic spondylodiscitis show variable tissue responses. CRP was best correlated with soft tissue changes, while ESR showed the best association with bony changes.

## Background

MRI is the gold-standard imaging method in the diagnosis of spondylodiscitis, given its high sensitivity and specificity [1–4]. The characteristic MRI findings of spondylodiscitis (upon the bone, disc, and surrounding soft tissues including the epidural space) have been described previously. Some of these features include edema in the disc or vertebral body, erosions or destruction of the vertebral endplate, and paraspinal or epidural inflammation or abscess [2–6]. The diagnosis of spondylodiscitis is usually based on clinical, laboratory, and radiological features. However, a definite diagnosis can only be made by microscopic or bacteriological examination of the infected tissues [7].

The management of spondylodiscitis is focused on eradicating the infection with antibiotic therapy. Clinical signs and symptoms, as well as laboratory data, including C-reactive protein (CRP) and erythrocyte sedimentation rate (ESR), are typically used for disease monitoring. The appropriate discontinuation of antibiotic therapy depends on symptom improvement or resolution, as well as ESR or CRP normalization [8]. The resolution of soft tissue changes and fatty deposition in the bone marrow are reliable signs of healing on MRI [9]. However, role of follow-up imaging is debated. There can be discrepancies with regard to the significance of bony or soft tissue responses to treatment. Some groups suggest that the soft tissue findings are more reliable markers of therapeutic response than are bony changes [9–11].

The current management algorithm for spondylodiscitis relies on a patient’s symptoms and laboratory findings as therapeutic response markers [2]. However, symptoms are non-specific and subjective. Further, laboratory markers reflect systemic status and are not specific to the location of the infection. In this regards, imaging can play a role in assessing the therapeutic response of infection site. Therefore, knowledge regarding follow-up MRI findings, together with the relationship between imaging and laboratory finding, might be helpful in judging therapeutic response for spondylodiscitis.

The purpose of this study was to test the hypothesis that MRI changes in the bone, soft tissue, and disc on follow-up imaging correlate with laboratory findings of treatment response.

## Methods

### Study population

This retrospective study was approved by the institutional review board at our institution. Between September 2006 and December 2014, patients who were diagnosed with pyogenic spondylodiscitis who had undergone baseline and follow-up MRI were selected. At our hospital, the policy of when to follow up MRI is not strictly established. Some infection specialists performed follow up MRI before patient discharge after antibiotic treatment as a comparative study regardless of symptoms while others ordered only when clinically aggravation is suspected. Patients were excluded if they had a history of tuberculosis spondylitis, had undergone surgery, or had an infection after spine surgery. A total of 48 patients (24 men and 24 women, mean age 60.9 years) were included.

### MRI protocol and imaging analysis

MRI was performed with 1.5-T (Magnetom vision [Siemens, Erlangen, Germany]) or 3.0-T (Trio Tim [Siemens, Erlangen, Germany], and Achieva TX [Philips Medical Systems, Eindhoven, The Netherlands]) scanners. The imaging sequences included the T1-weighted and T2-weighted images for the axial and sagittal planes and contrast-enhanced T1-weighted fat suppressed axial and sagittal planes. Nine baseline MRIs were obtained from other institutions. Contrast enhancement was not performed in six baseline studies including four exams from other institution. Two musculoskeletal radiologists reviewed the pair of baseline and follow-up MRIs in consensus. The radiologists were blinded to other clinical information. The bone findings were assessed according to the volume of bone marrow enhancement or edema. The cases were separated into three subgroups (< 33%, 33–66%, ≥66%) based on the baseline MRI. The follow-up MRIs were graded as improved, equivocal, or worse (from baseline). In case of multi-level involvement, most severely involved level was assessed. The disc height of the involved segment was compared between the baseline and follow-up MRIs, and graded as equivocal or worse. The soft tissue findings were determined as follows. Improved meant that there was decreased signal abnormality or abscess size in the paraspinal/psoas muscle, and/or epidural space compared with the baseline imaging findings. Equivocal results indicated that some areas improved while others worsened, or there was no substantial change. Worse findings reflected increased signal abnormality or abscess size in the paraspinal/psoas muscle, and/or epidural space or new levels involved, compared with the baseline imaging findings [10]. Comparisons were primarily made based on the contrast-enhanced images. In cases of unavailable contrast-enhanced images, non-enhanced T2-weighted and T1-weighted images were reviewed.

### Laboratory finding

**Fig. 1:**
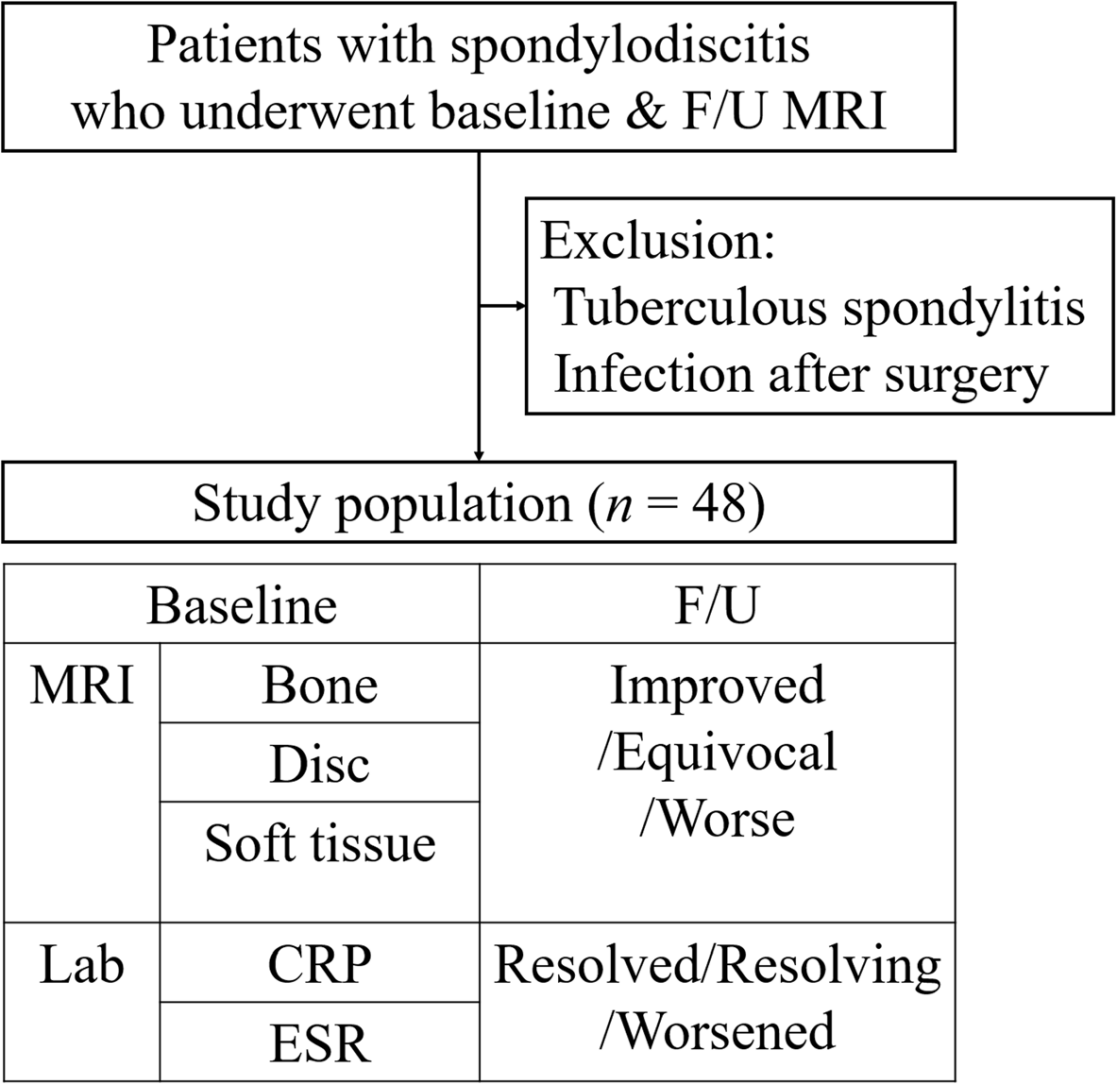
A diagrammatic flow chart of the study

The available medical records of the baseline and follow-up ESR and CRP levels were obtained. The values of the closest date with MRI examination were selected. Regarding our protocol of laboratory tests, ESR and CRP were routinely monitored after approximately 4 weeks of antimicrobial therapy. Three groups were established based on follow-up laboratory values: normalized (resolved); decreased but not normalized (resolving); and increased (worsened). Figure 1 illustrates the study design.

### Statistical analysis

The relationship between changes in the laboratory findings and changes in the MRI findings were analyzed using the Spearmann correlation test. Statistical analyses were performed using SPSS version 20.0 (SPSS Inc., Chicago, IL). *P* values < 0.05 were considered statistically significant.

## Results

### Subject characteristics

In our subject group, the mean period between the baseline and follow-up MRIs was 42.25 (± 22.25) days. The pathogens involved in the spondylodiscitis were identified as follows: culture-negative (*n* = 22), *Staphylococcus aureus* (*n* = 12), *S. epidermidis* (*n* = 7), methicillin-resistant *S. epidermidis* (n = 1), *Klebsiella pneumoniae* (*n* = 3), *Escherichia coli* (n = 2), and *Corynebacterium striatum* (*n* = 1). Eighteen cases were confirmed by blood culture and eight cases were confirmed by targeted biopsy or aspiration. The involved spinal levels included the cervical spine (n = 1), thoracic (*n* = 5), thoracolumbar (*n* = 4), lumbar (*n* = 34), and lumbosacral (n = 4). CRP resolved in 19 cases (1.9 ± 1.4 mg/L), and worsened in 10 cases (83.8 ± 76.7 mg/L). ESR improved in eight cases (8.0 ± 4.9 mm/hr), and worsened in 18 cases (57.5 ± 21.3 mm/hr). Eight cases showed improvement in both CRP and ESR, while five cases showed worsening of both CRP and ESR. The mean intervals between the laboratory examinations and MRI were 2.69 days for CRP and 2.73 days for ESR in the baseline studies, and 3.1 and 1.83 days on follow-up examinations, respectively.

### Correlation between follow-up MRI findings and CRP-based subgroups

**Fig. 2:**
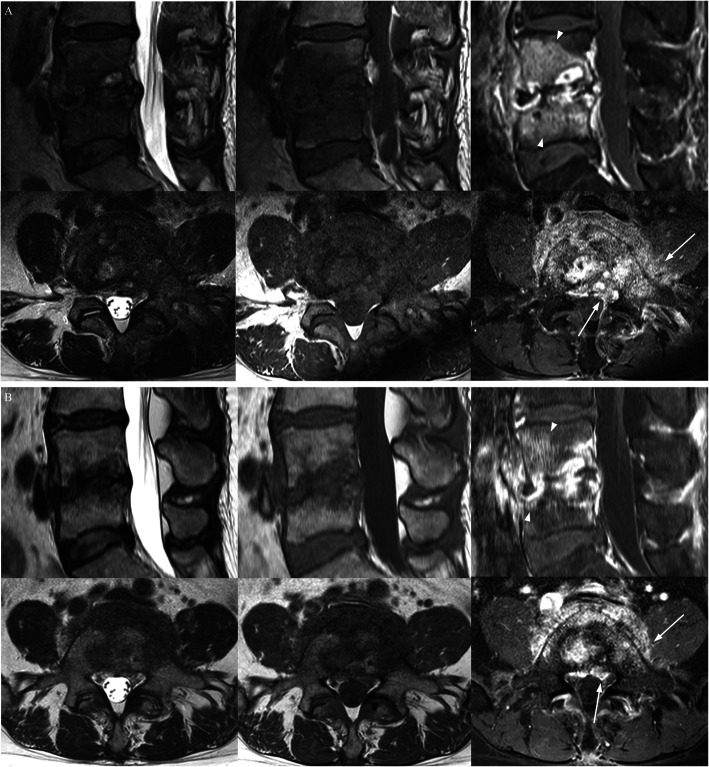
A patient with spondylodiscitis at L4–5 (culture (−), F/U interval 55 days). T2-weighted (left), T1-weighted (middle), and contrast-enhanced fat suppressed T1-weighted images (right) of the spine. Comparing contrast-enhanced fat suppressed T1-weighted sagittal (upper) and axial (lower) images of baseline (**a**) and follow-up (**b**), osteomyelitis (arrow heads) and soft tissue inflammation (arrows) were improved, while disc height worsened. Both CRP and ESR were resolved at follow-up

**Fig. 3:**
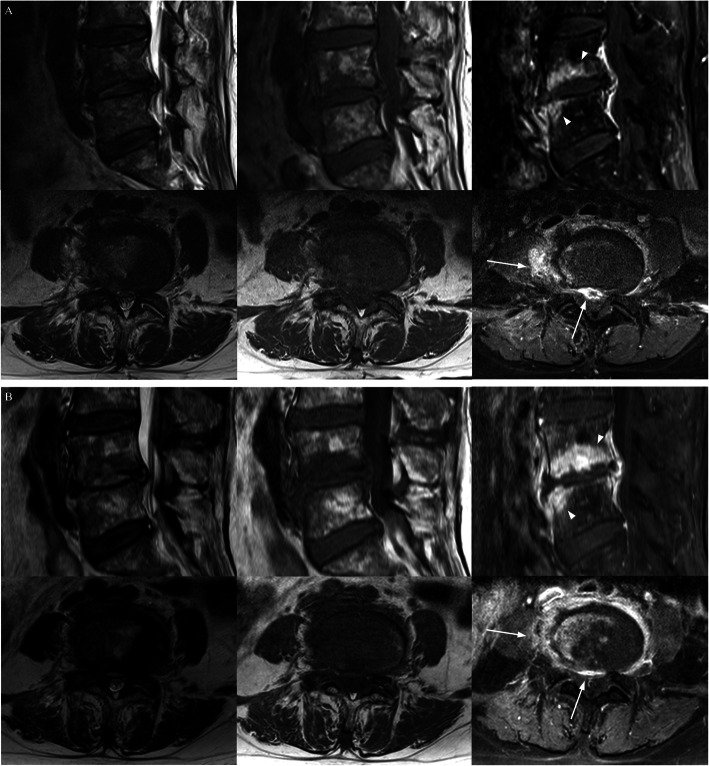
A patient with spondylodiscitis at L4–5 (culture (−), follow-up interval 44 days). T2-weighted (left), T1-weighted (middle), and contrast-enhanced fat suppressed T1-weighted images (right) of the spine. Comparing contrast-enhanced fat suppressed T1-weighted sagittal (upper) and axial (lower) images of baseline (**a**) and follow-up (**b**), bone findings worsened (arrow heads) and soft tissue findings improved (arrows). The disc height change was equivocal. CRP was resolved (24.4 to 3.41 mg/L [0.0–5.0]) and ESR was resolving (44 to 39 mm/hr. [0–15]) at baseline and follow-up, respectively

Based on the CRP results, the number of subjects in the resolved, resolving, and worsened group were 19, 19, and 10, respectively. The bone findings worsened (6 of 19) or were equivocal (6 of 19) in the resolved group. In contrast, the soft tissue findings were improved (14 of 19) in many of the cases in the resolved group (Figs. 2 and 3). The disc findings did not improve in any of the subjects. Table 1 demonstrates changes on follow-up MRI findings in CRP-based subgroups. CRP was significantly correlated with changes in bone and soft tissue (*p* = 0.004 and 0.001, respectively), but not with changes in disc height (*p* = 0.072). This correlation was best with soft tissue changes (rho: 0.482), followed by bony changes (rho: 0.408).

**Table 1:** Correlation between follow-up MRI findings and CRP-based subgroups

	Resolved group (*n* = 19)	Resolving group (*n* = 19)	Worsened group (*n* = 10)
Bone			
Improved	7	1	0
Equivocal	6	5	3
Worse	6	13	7
Disc			
Equivocal	13	7	4
Worse	6	12	6
Soft tissue			
Improved	14	5	2
Equivocal	2	7	1
Worse	3	7	7

### Correlation between follow-up MRI findings and ESR-based subgroups

**Fig. 4:**
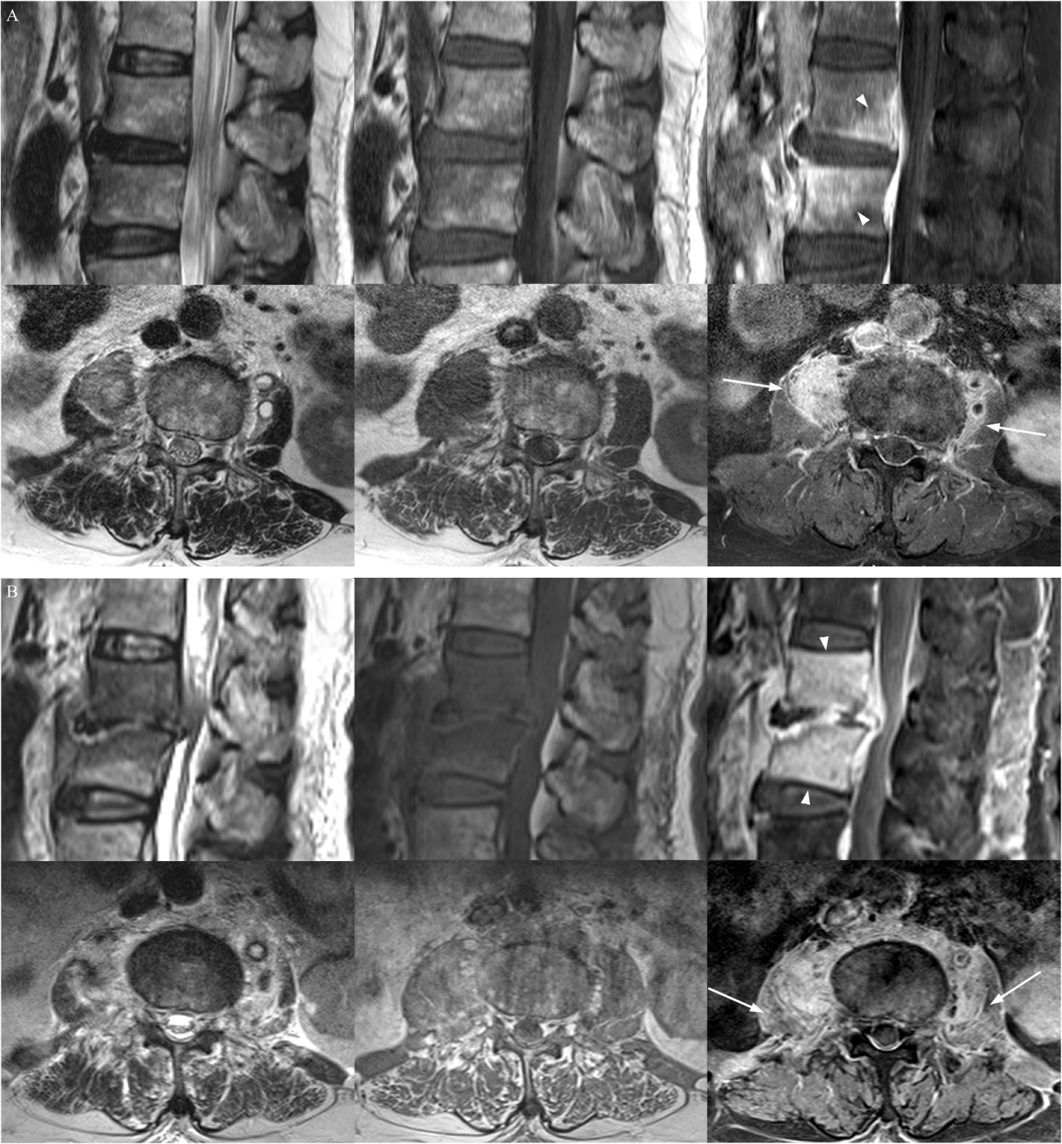
A patient with spondylodiscitis at L2–3 (*Klebsiella pneumoniae*, follow-up interval 40 days). T2-weighted (left), T1-weighted (middle), and contrast-enhanced fat suppressed T1-weighted images (right) of the spine. Comparing contrast-enhanced fat suppressed T1-weighted sagittal (upper) and axial (lower) images of baseline (**a**) and follow-up (**b**), bone (arrow heads), soft tissue (arrows), and disc findings all worsened. CRP was resolving (130.9 to 57.5 mg/L [0.0–5.0]) and ESR was worsen (52 to 68 mm/hr. [0–15]) at baseline and follow-up, respectively

Based on the ESR results, the numbers of subjects in the resolved, resolving, and worsened group were 8, 22, and 18, respectively. The bone findings improved (3 of 8) or were equivocal (4 of 8) in the resolved subjects, while they were equivocal (7 of 22) or worse (11 of 22) in the resolving group (Figs. 3 and 4). The soft tissue findings improved in 50% (4 of 8) of the resolved group and 63.6% (14 of 22) of the resolving group. The disc findings, like in the CRP-based analysis, were not improved in all subjects. Table 2 demonstrates changes in follow-up MRI findings in the ESR-based subgroups. Changes in the bone and soft tissue both showed significant correlation in the ESR-based subgroups (*p* = 0.001 and 0.007, respectively), but not with the disc changes (*p* = 0.507). The correlation was better with the bony changes (rho: 0.447) than with the soft tissue changes (rho: 0.387).

**Table 2:** Correlation between follow-up MRI findings and ESR-based subgroups

	Resolved group (*n* = 8)	Resolving group (*n* = 22)	Worsened group (*n* = 18)
Bone			
Improved	3	4	1
Equivocal	4	7	3
Worse	1	11	14
Disc			
Equivocal	6	9	9
Worse	2	13	9
Soft tissue			
Improved	4	14	3
Equivocal	2	4	4
Worse	2	4	11

## Discussion

In this study, we evaluated the relationship between follow-up MRI findings of specific tissues and laboratory results in pyogenic spondylodiscitis. Follow-up MRI findings may demonstrate variable responses according to the tissue. The CRP level was best correlated with changes in the soft tissue, while ESR had the highest association with bony changes.

The treatment of spondylodiscitis is focused on eradicating the infection, and restoring or preserving the spinal structure and stability [2, 7]. Monitoring spondylodiscitis can be complex, because the diagnosis is based on clinical, laboratory, and radiologic information. A clinical practice guideline of the Infectious Disease Society of America recommends monitoring systemic inflammatory markers after 4 weeks of antimicrobial therapy [12]. Unchanged or increasing values should increase the suspicion for treatment failure. However, follow-up imaging is not routinely recommended for a patient with favorable clinical and laboratory response, but is selectively recommended in those with poor clinical response [12–14]. The criteria for discontinuing antimicrobial treatment includes symptom resolution or improvement and normalization of ESR or CRP [8].

Although clinical symptoms are an important factor, they can be subjective and non-specific. Therefore, laboratory results may be more reliable and objective criteria. ESR and CRP are useful inflammatory markers for diagnosing and monitoring infections [12, 15, 16]. The ESR is affected by increasing concentrations of fibrinogen, the main clotting protein, during an inflammatory reaction. In contrast, CRP is primarily produced by the liver in response to cytokines such as interleukin-1 [17, 18]. CRP has a rapid response to inflammation due to its short half-life, compared to a slow response of ESR [18]. In the early phase of an infection or inflammation, CRP can rise before ESR does. In contrast, in the resolving phase, CRP normalizes while ESR remains elevated. Therefore, CRP is a more sensitive and specific acute phase reactant, and is also more responsive to changes in the patient’s condition than is ESR. In patients with acute spondylodiscitis, elevated CRP values returned to normal within 3 months of successful treatment [7, 18]. However, in the case of detecting low-grade bony or joint infections, or autoimmune diseases such as systemic lupus erythematous, ESR may be a better marker [18]. In spondylodiscitis, ESR is elevated in over 90% of cases regardless of severity of infection or the patient’s age. A reduction in ESR below 25% of its presenting value is known to be a good prognostic marker [7].

Several previous studies have examined the findings and roles of follow-up MRI in spondylodiscitis [9–11, 19–22]. Although MRI is currently the imaging modality of choice for evaluating spondylodiscitis, its role in follow-up surveillance has not been established [12]. Numaguchi et al. described that persistent enhancement can be seen in both the bone and disc despite clinical improvement [20]. Gillams et al. reported progression in bone or disc changes despite clinical improvement [9]. Kowalski et al. reported that soft tissue findings, rather than bone findings, on follow-up MRI are related to clinical status based on symptoms and sign [11]. Kowalski et al. suggested that a patient’s clinical status and inflammatory biomarker response would be helpful for selecting patients at high risk of treatment failure who may require follow-up MRI [10]. However, neither of these markers is ideal. Clinical symptoms can be subjective, while laboratory results are not specific to the location of the infection. Furthermore, there may be a discrepancy between tests. In terms of cost analysis on routine follow-up MRI in patients with pyogenic spondylodiscitis, clinical findings and laboratory markers may be satisfactory to monitor the therapeutic response. However, laboratory markers are systemic, so local problem needs to be checked with MRI complementarily along with clinical symptoms. To the best of our knowledge, this is the first study that compares imaging findings with laboratory results in spondylodiscitis. Knowledge of this relationship might be helpful not only in deciding the necessity of follow-up MRI, but also in interpreting follow-up MRIs.

In our study, CRP was best correlated with soft tissue changes, while ESR was best correlated with bone changes. The mechanism for this relationship is not clear. It might be related with the rapid improvement in CRP and soft tissue findings, compared to those in ESR and bone. The persistent bone signal change in MRI despite the clinical improvement might be related with the abundant vascular supply or increasing granulation tissue in bone [11]. The slow healing of bone compared to in soft tissue, and the relatively longer half-life of ESR (compared to that of CRP) may have influenced our results. The disc height never improved in our study, and was thought to be a sequelae of spondylodiscitis regardless of disease progression or improvement. In some studies, authors evaluated the disc signal change or enhancement [9, 11, 20]. However, we did not assess disc signal change due to the wide observer variability in our preliminary evaluation.

It is not clearly established how to follow MRI after the treatment of spondylodiscitis, but routine follow-up of MRI is not recommended [12]. In case of follow-up MRI due to clinical issue such as back pain or radiculopathy without laboratory abnormality, signal change especially in bone can be interpreted carefully because it may be a slow response to a favorable therapy. If soft tissue findings are improving or improved, they are favorable responses. In case of laboratory abnormality, however, MRI can be required to differentiate the condition as unfavorable treatment response or problem other than spine. Soft tissue findings seem to be more reliable than bone findings to judge the response to therapy [11].

There are several limitations in this study. First, we only observed the relationship between laboratory findings and imaging findings without considering clinical decision for therapeutic response which might change the treatment plan. In our study population, additionally, there were no cases which antibiotic regimen changed or surgical treatment performed as regarded progressive disease although both ESR and CRP at the time of follow-up MRI were resolving or resolved. Second, the sample size of patients was small. Therefore, a larger population study is required to validate our results. The third is the narrow inclusion criteria. We only included patients with medical therapy who underwent follow-up MRI due to analysis difficulties on post-operative images, which were caused by artifacts. This method may have restricted our results to relatively mild forms of spondylodiscitis. The fourth limitation is that there was a time interval between the imaging and laboratory results. Although we examined laboratory results from the closest date with MRI, we observed relatively rapid changes in the laboratory results (and especially in CRP). These changes may have changed the subgroups (e.g. resolving to resolved group) by even a few day intervals. Furthermore, we did not account for differences caused by the etiologic pathogens. Different pathogens and antibiotics may alter the clinical course; however, due to the small number of subjects, we could not consider the differences caused by the pathogens and antibiotics. Finally, we analyzed the MRI using a consensus manner without assessing inter-observer agreement.

## Conclusion

Follow-up MRI findings of pyogenic spondylodiscitis may show variable tissue responses. CRP was best correlated with soft tissue changes, while ESR had the greatest association with bony change. Knowledge regarding these relationships may be helpful in deciding or interpreting follow-up imaging. Like ESR normalizes more slowly than CRP, bone abnormalities on MRI takes more time to be normalized than soft tissue abnormalities. If ESR or CRP increases, follow-up MRI can be required to differentiate the condition is resulted from treatment failure or problem other than spondylodiscitis.

## Data Availability

The datasets used and/or analyzed during the current study are available from the corresponding author upon reasonable request.

## Abbreviations

CRP: C-reactive protein
ESR: erythrocyte sedimentation rate
MRI: Magnetic resonance imaging
SPSS: Statistic Package for Social Science
